# A new lysozyme from the eastern oyster, *Crassostrea virginica*, and a possible evolutionary pathway for i-type lysozymes in bivalves from host defense to digestion

**DOI:** 10.1186/1471-2148-10-213

**Published:** 2010-07-15

**Authors:** Qinggang Xue, Michael E Hellberg, Kevin L Schey, Naoki Itoh, Ron I Eytan, Richard K Cooper, Jerome F La Peyre

**Affiliations:** 1 Department of Veterinary Science, Louisiana State University Agricultural Center, Baton Rouge, LA 70830, USA; 2 Department of Biological Sciences, Louisiana State University, Baton Rouge, LA 70803, USA; 3 Department of Cell and Molecular Pharmacology, Medical University of South Carolina, Charleston, SC 29425, USA; 4 Mass Spectrometry Center, Department of Biochemistry, Vanderbilt University, Nashville, TN 37240, USA; 5 Graduate School of Agricultural Science, Tohoku University, 1-1 Tsutsumidori Amamiya-machi, Aoba-ku, Sendai 981-8555 Miyagi, Japan

## Abstract

**Background:**

Lysozymes are enzymes that lyse bacterial cell walls, an activity widely used for host defense but also modified in some instances for digestion. The biochemical and evolutionary changes between these different functional forms has been well-studied in the c-type lysozymes of vertebrates, but less so in the i-type lysozymes prevalent in most invertebrate animals. Some bivalve molluscs possess both defensive and digestive lysozymes.

**Results:**

We report a third lysozyme from the oyster *Crassostrea virginica*, cv-lysozyme 3. The chemical properties of cv-lysozyme 3 (including molecular weight, isoelectric point, basic amino acid residue number, and predicted protease cutting sites) suggest it represents a transitional form between lysozymes used for digestion and immunity. The cv-lysozyme 3 protein inhibited the growth of bacteria (consistent with a defensive function), but semi-quantitative RT-PCR suggested the gene was expressed mainly in digestive glands. Purified cv-lysozyme 3 expressed maximum muramidase activity within a range of pH (7.0 and 8.0) and ionic strength (I = 0.005-0.01) unfavorable for either cv-lysozyme 1 or cv-lysozyme 2 activities. The topology of a phylogenetic analysis of cv-lysozyme 3 cDNA (full length 663 bp, encoding an open reading frame of 187 amino acids) is also consistent with a transitional condition, as cv-lysozyme 3 falls at the base of a monophyletic clade of bivalve lysozymes identified from digestive glands. Rates of nonsynonymous substitution are significantly high at the base of this clade, consistent with an episode of positive selection associated with the functional transition from defense to digestion.

**Conclusion:**

The pattern of molecular evolution accompanying the shift from defensive to digestive function in the i-type lysozymes of bivalves parallels those seen for c-type lysozymes in mammals and suggests that the lysozyme paralogs that enhance the range of physiological conditions for lysozyme activity may provide stepping stones between defensive and digestive forms.

## Background

Lysozymes are ubiquitous antibacterial enzymes that lyse bacterial cell walls [1]. Lysozymes identified from organisms ranging from bacteriophage to humans share a muramidase activity cleaving the glycosidic bond between N-acetylmuramic acid and N-acetylglucosamine of peptidoglycan, a major component of the bacterial cell wall [2-4], but have different amino acid sequences and biochemical properties. Groups within the lysozyme superfamily have been classified as phage-type lysozymes, plant lysozymes, i- (invertebrate) type, c- (chicken) type, and g- (goose) type lysozymes [1,5-10]. Structural studies reveal that a phylogenetically broad sample of lysozymes (bacteriophage T4 lysozyme, i-type Japanese littleneck clam (*Tapes japonica *or *Venerupis philippinarum *in the NCBI Taxonomy database) lysozyme, c-type chicken egg-white lysozyme, and the g-type goose egg-white lysozyme) all have a similar conformation surrounding the enzyme catalytic center, even though their amino acid sequences bear little similarity [11-14]. Given their shared activities and structure, these different lysozymes appear to have been derived from a common ancestor [11-13].

The principal function attributed to lysozymes in most animals is host defense [15]. Lysozymes break down the bacterial cell wall through the muramidase activity, resulting in the eventual lysis of bacterial cells [16]. In addition, soluble fragments released by lysozyme degradation of peptidoglycan may play a role in immunomodulation in both vertebrates and invertebrates [17-19]. Moreover, lysozymes can also exert antimicrobial activity against bacteria and viruses through a mechanism independent of their muramidase activity [20-28].

In addition to a role in host defense, some c-type lysozymes have developed a second function that also makes use of their ability to break down bacterial cell walls: digestion. Lysozymes play a role in digestion in ruminants [29,30], in leaf-eating monkeys [31], in sloths [32], in a leaf-eating bird [33], and in a variety of arthropods that feed on bacteria [34-41]. To function as a digestive enzyme, lysozymes must be expressed at high concentration in the stomach and function in the highly acidic and protease-rich environment of that organ [10,42]. In primates, the amino acid replacements that changed lysozyme host defense function to a digestive function have been the result of convergent evolution through positive selection [31,43,44].

The addition of a digestive function to a host defense function in c-type lysozymes has been paralleled in i-type lysozymes, the invertebrate type first identified in the sea star *Asteria rubens *[45,46]. In bivalve molluscs, bacteria are known to constitute a significant portion of the diet [47], and a digestive function for lysozyme has been suggested in many studies [9,48-55]. In some of these cases, two lysozyme paralogs co-occur in the same taxon, one expressed in the gills and mantle and potentially maintaining the ancestral host defense function, and the other found in digestive gland and presumably adaptively differentiated for digestive function. The most fully characterized of these paralogs are two lysozymes, cv-lysozyme 1 and cv-lysozyme 2, found in the eastern oyster, *Crassostrea virginica *[56-58]. Cv-lysozyme-1, a 17,861 Da protein originally purified from oyster hemolymph, has optimal muramidase activity at high ionic strength and a relatively broad pH range [56]. The major sites of expression of cv-lysozyme 1 (mantle and gills), its abundance in hemolymph, and its strong antimicrobial activity all suggest that its main role is in host defense [58]. On the other hand, cv-lysozyme 2, a 12,984.6 Da protein expressed in the basophil cells of digestive tubules, was purified from crystalline styles and digestive glands, suggesting a role in digestion [57]. While cv-lysozyme 2 shows high amino acid sequence similarity to other bivalve mollusc i-type lysozymes, including cv-lysozyme 1, it has a lower isoelectric point and fewer protease-cutting sites in its amino acid sequence. These differences resemble adaptive changes found in vertebrate c-type lysozymes that have switched from host defense to digestive functions [10,42].

While we have previously proposed that i-type lysozymes may have followed a comparable adaptive path between host defense and digestion as seen in some vertebrate c-type lysozymes [57], the chain of evolutionary steps involved in the shift between functions remains unknown. Here, we characterize a third lysozyme from the eastern oyster, designated cv-lysozyme-3, that reveals some of these steps. The objectives of this research were: 1) to characterize the biochemical and antimicrobial properties of this new lysozyme, 2) to determine its cDNA and amino acid sequence and where this gene is expressed, 3) to analyze its phylogenetic relationship with the two other eastern oyster lysozymes (i.e., cv-lysozyme 1 and cv-lysozyme 2) as well as with i-type lysozymes from other bivalve species, and 4) to test whether selective changes to the protein accompanied the functional shift between defensive and digestive lysozymes in *C. virginica*.

## Results

### Cv-lysozyme 3 purification

A protein with high lysozyme activity but differing in optimal conditions for the activity from cv-lysozyme 1 and cv-lysozyme 2 was purified from *Crassostrea virginica *shell liquor using a combination of ion exchange and size exclusion chromatographies, hydrophobic interaction chromatography (HIC) and reverse phase HPLC. We used "lysozyme activity" to refer the *M. lysodeikticus* peptidoglycan degrading or muramidase activity in this research. From the 126.8 g of total proteins prepared from 20 L of shell liquor, 50.7 mg of proteins with high lysozyme activity were obtained after fractionation by chromatographies. HIC separated these proteins into 2 major absorbance peaks, both containing lysozyme activity. After reverse phase HPLC purification of the proteins in the second HIC absorbance peak, a protein collected from the fraction in a major absorbance peak at the retention time of 35.4 min showed high lysozyme activity and was therefore designated cv-lysozyme 3. A total of 0.45 mg of cv-lysozyme 3 was eventually purified.

**Figure 1:**
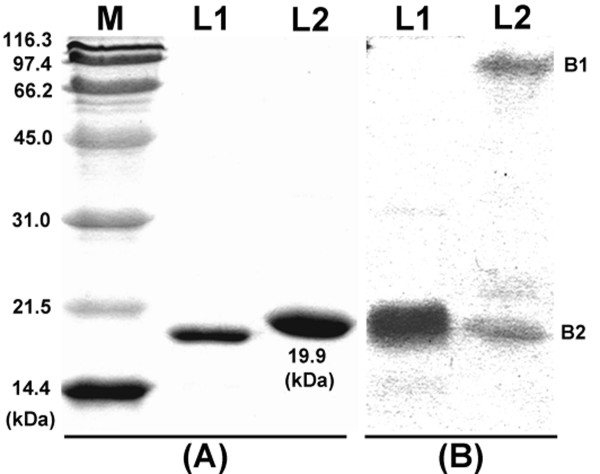
**SDS-PAGE of purified cv-lysozyme 3**. Electrophoresis was carried out in 12.5% acrylamide/bis gel under reduced (A) and non-reduced (B) conditions and the gel was then stained with Coomassie blue R-250. M. protein standards with the molecular sizes indicated; L1: purified cv-lysozyme 1; L2: purified lysozyme 3, detected as a single band with a molecular size of around 19.9 kDa under reduced conditions (A), two bands under non-reduced conditions (B).

**Figure 2:**
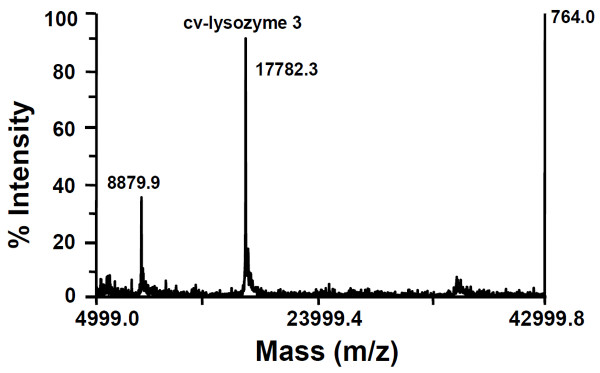
**MALDI mass spectrum of purified cv-lysozyme 3**. Cv-lysozyme 3 was detected as an MH+ ion at m/z 17782.3. The signal at m/z 8879.9 represents the doubly charged intact cv-lysozyme 3 molecule.

Purified cv-lysozyme 3 appeared as a single band with a molecular size of 19.9 kDa as determined by SDS-PAGE under reducing conditions (Figure 1A). However, under non-reducing conditions, a second band with a molecular size of >100 kDa was also detected (Figure 1B). It appeared that the high molecular band represented a cv-lysozyme 3 polymer formed via electrostatic force, a phenomenon reported for other lysozymes [14,59-62]. MALDI mass spectrometry revealed an ion of 17782.3 Da for the purified cv-lysozyme 3 (Figure 2). The MALDI measured molecular weight of the reduced and alkylated cv-lysozyme 3 was 18939 (not shown), indicating the presence of 20 cysteine residues.

### pH and ionic strength conditions for optimal lysozyme activity

**Figure 3:**
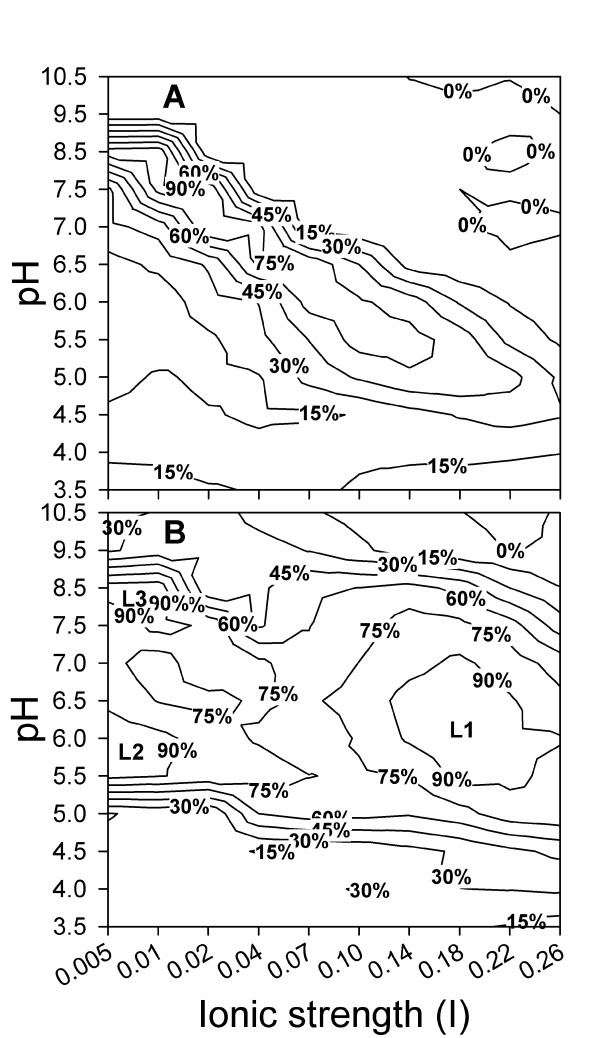
**pH and ionic strength conditions for optimal lysozyme activity of purified cv-lysozyme 3**. Optimal pH and ionic strength were determined by measuring cv-lysozyme 3 degrading activity against a *Micrococcus lysodeikticus *cell wall suspended in 120 buffers covering a pH range of 3.5-10.5 and ionic strength range of I = 0.005-0.260 at 25°C (A). The conditions were compared with that of cv-lysozyme 1 [56] and cv-lysozyme 2 [57] by superimposing the optimal ionic and pH conditions of all the 3 *C. virginica *lysozymes; L1, L2, and L3 indicate the optimal activity conditions for cv-lysozyme 1, cv-lysozyme 2, and cv-lysozyme 3 respectively (B). Shown were percent maximum activities of each lysozyme.

Purified cv-lysozyme 3 expressed more than 90% of its maximum activity in the pH range of 7.5-8.5 and the ionic strength range of I = 0.005-0.03 (Figure 3A). Cv-lysozyme 3 retained high **lysozyme activity **in conditions with a combination of higher pH and lower ionic strength or a combination of lower pH and higher ionic strength. In buffers with pH 7.5, for example, cv-lysozyme 3 retained more than 60% of its maximum activity in an ionic strength range of I = 0.01-0.04. In contrast, when the pH was 5.5 the similar activity level was detected in the ionic strength range of I = 0.10-0.14 (Figure 3A). Superimposing the optimal activity conditions of cv-lysozyme 3 (Figure 3A) with those of cv-lysozyme 1 [57] and cv-lysozyme 2 [58] indicated that cv-lysozyme 3 exerted optimal lysozyme activity under the pH and ionic strength conditions that differ from that optimal for the other two lysozymes (Figure 3B).

### Antibacterial activities

Purified cv-lysozyme 3 at concentration of 12.5 μg/mL significantly inhibited the growth of *E. coli*. However, no significant inhibition activity against *V. vulnificus *was detected at the highest cv-lysozyme 3 concentration tested (i.e., 100 μg/mL). Cv-lysozyme 3 possessed an antibacterial activity against *E. coli *stronger than cv-lysozyme 2 but weaker than cv-lysozyme 1 and both cv-lysozyme 3 and cv-lysozyme 2 have limited effect againt *V. vulnificus *(Table 1).

**Table 1:** Properties of eastern oyster lysozymes.

Characteristic	cv-lysozyme-3	cv-lysozyme-1*	cv-lysozyme-2**
Lysozyme activity			
Optimal pH	7.5-8.5	5.5-6.5	5.5-6.5
Optimal ionic strength (I)	0.005-0.01	0.18-0.20	0.005-0.01
Antibacterial activity (MIC)			
*E. coli*	12.5 μg/mL	1.56 μg/mL	50 μg/mL
*V. vulnificus*	ND***	12.5 μg/mL	100 μg/mL
Molecular properties			
MW (Dalton)^b^	17782.3	17861.0	12984.6
Theoretical pI	6.84 (7.12)	8.95	6.33
Amino acid sequence			
Total amino acid number	168	164	117
Arginine residue number	5	19	5
Protease cutting sites			
Trypsin	9	18	9
Thermolysin	32	28	20
Pepsin (pH > 2.0)	19	12	5
Major sites of gene expression	Digestive glands	Labial palps, mantle	Digestive glands
Tissues with low mRNA level	Mantle, labial palps, gill, style sac-midgut, hemocytes	Gill, style sac-midgut, digestive gland, gonad	Style sac-midgut

*[56,58]; ** [57]; *** Not detected at 100 μg/mL

The biochemical and molecular characteristics of three mature proteins with lysozyme activities identified from the eastern oyster, *Crassostrea virginica*, were compared.

### Cv-lysozyme 3 amino acid sequence

The 15 N-terminal amino acid residues of cv-lysozyme 3 determined by Edman degradation were Ser-Asp-Ala-Pro-Cys-Thr-Asn-Ser-Gly-Gly-Val-Cys-Gln-Asp-Asp. In addition, the amino acid sequences of 9 peptides derived from trypsin treatments of purified cv-lysozyme 3 was determined by tandem mass spectrometry (Table 2). The mass spectrometry-determined sequence identified 167 of the 169 amino acid residues deduced from the cDNA sequence, with the Arg36 and the C-terminal Lys of the deduced sequence missing. Given the finding that purified cv-lysozyme 3 was 147.3 Da smaller than the cDNA predicted molecule, the C-terminal Lys was likely removed during posttranslational modification of the protein. The protein sequence data reported in this paper will appear in the Uniprot Knowledgebase under the accession number P85518.

**Table 2:** Peptides sequenced by tandem mass spectrometry

Residues	Predicted MW*	Observed MW	Sequence
1-35	3796.5	3797.3	SDAPCTNSGGVCQDDHLACHNGHYQSGLCTGGAHR
37-69	3736.6	3737.4	CCLTSASHTGSFSTGIVSQQCLQCICNVESGCK
70-88	2218.0	2218.5	AIGCHFDVNSDSCGYFQIK
89-103	1780.7	1781.2	EGYWHDCGSPGSSWR
104-114	1196.5	1195.9	SCANDLACASK
115-122	1014.4	1014.5	CVQAYMSR
123-138	1880.8	1881.3	YIGFSGCSHSCESYAR
139-148	1010.5	1010.5	IHNGGPAGCK
149-168	2293.1	2293.6	HTNTLGYWSHVHAQGCSHNS

*Predicted monoisotopic masses

### Cv-lysozyme 3 cDNA sequence

A 663-bp cDNA sequence was identified after 5'- and 3'- RACE reactions. The first ATG codon of the sequence at position 50-52 was assigned as the translational initiation codon and the TAA codon at position 611-613 as the termination codon. Based on this assignment, a 564 bp open reading frame encoding 187 amino acid residues was determined. SignalP 3.0 predicted that the N-terminal 18 amino acid residues of the deduced sequence constituted a signal peptide. After exclusion of the predicted signal peptide, the calculated molecular weight of the cv-lysozyme 3 protein was 17929.6 Da and the isoelectric point 7.05. Comparison of the amino acid sequence of cv-lysozyme 3 and that of the two other eastern oyster lysozymes detected a 68% identity and an 82% similarity with cv-lysozyme 1 and a 64% identity and a 78% similarity with cv-lysozyme 2. The cDNA sequence and the deduced amino acid sequence were registered respectively in the GenBank under accession numbers AB427186 and BAG41979.

### Detection of cv-lysozyme 3 gene expression by semi-quantitative RT-PCR

**Figure 4:**
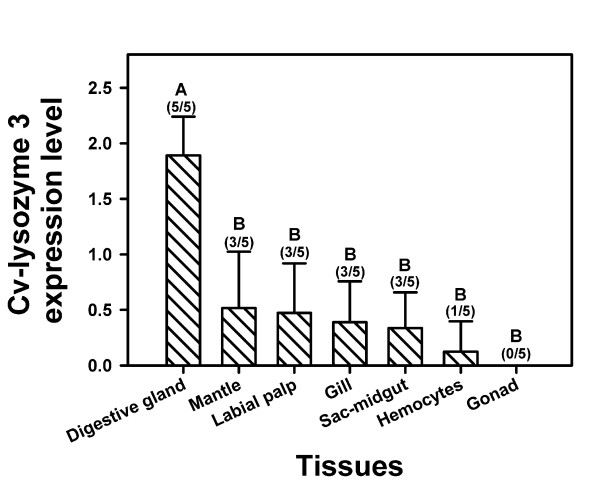
**Tissue-specificity of cv-lysozyme 3 gene expression determined using semi-quantitative RT-PCR**. Expression levels of cv-lysozyme 3 mRNA (based on comparison between the cv-lysozyme 3 amplicons generated in 35 PCR cycles and the 28 S rRNA amplicons generated in 25 PCR cycles for sample normalization) are indicated for each tissue tested. Mean ± standard deviation (SD) are shown for each tissue, with fractions indicating the proportion of the 5 oysters assayed in which cv-lysozyme 3 mRNA was detected. Columns with the same letter are not significantly different in expression level (P < 0.05).

Semi-quantitative RT-PCR amplified the specific cv-lysozyme 3 cDNA fragment from digestive tissue, mantle, labial palps, gills, style-midgut sac, and hemocytes (Figure 4). However, the expression level of digestive glands was 3.6-15.7 times that of the other tissues, with a mean expression level of 1.89 in digestive gland compared to 0.52 in mantle, 0.47 in labial palps, 0.39 in gills, 0.34 in sac-midgut, and 0.12 in hemocytes. In addition, cv-lysozyme 3 mRNA was detected in the digestive glands of all 5 oysters tested, compared to only 3 out of 5 in mantle, labial palps, gills, and style-midgut sac, and 1 out of 5 in hemocytes. No cv-lysozyme 3 mRNA was detected in the gonad of any of the 5 oysters analyzed. Cv-lysozyme 3 expression level differed statistically between digestive gland and the six other tissue tested (Figure 4). The specificity of quantitative RT-PCR was confirmed by sequencing the PCR products.

### Alignment and phylogenetic position of cv-lysozyme 3 relative to other bivalve lysozymes

**Figure 5:**
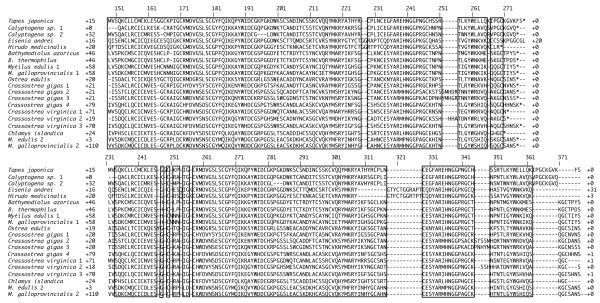
**Amino acid sequence alignment of the i-type lysozymes from bivalve molluscs and annelids**. Shown are the alignments of 20 known bivalve i-lysozymes, along with those of two annelids (*Eisenia andrei *and *Hirudo medicinalis*) based on ClustalW (top) and BAli-Phy (bottom) algorithms. Boxed sequences indicate the core part of the aligned proteins used for phylogenetic analysis. Residues in front of the position of the first intron are excluded, with the number of residues deleted indicated. The number of residues omitted from the C-terminus is also indicated. The catalytic residues occur at E163 and D174 in the ClustalW (top) alignment. Positional numbering is based on the full alignments.

The BAli-Phy alignment of the bivalve i-lysozymes includes long stretches of residues at the beginning and end of the proteins that are either species-specific or align with just a few other homologs (not shown). The total length of the gapped ClustalW alignment was 298 amino acid residues; for BAli-Phy, it was 410. The conserved core of the bivalve lysozymes, however, includes a long stretch that is aligned with few indels and with high probability (Figure 5). Residues included in the core differ between the alignments based on the two algorithms. The core ClustalW alignment includes 114 residues, the core Bali-Phy alignment 108. In both cases, the conserved core includes the two catalytic residues.

**Figure 6:**
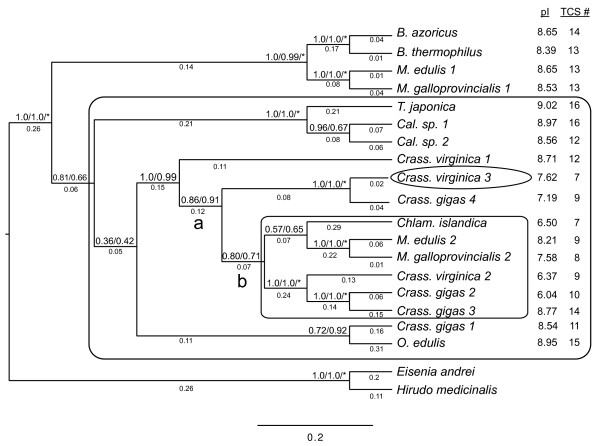
**Phylogenetic relationships among bivalve i-type lysozymes**. The phylogenetic tree was inferred based on a Bayesian analysis of amino acid sequences from the core alignment. Numbers above branches indicate Bayesian posterior probabilities, the first for the analysis based on the BAli-Phy alignment and the second for the analysis based on the ClustalW analysis. Asterisks indicate nodes supported by maximum likelihood bootstrap values ≥ 0.75. Branch lengths are shown below branches. The novel *C. virginica *lysozyme isolated here (cv-lysozyme 3) is circled. The larger box indicates the 13 sequences used for the PAML analysis. The smaller box indicates the clade containing the digestive cv-lysozyme 2 (cv2C). Isoelectric points (pI) and trypsin cutting site numbers (TCS#) predicted from the aligned core sequences are shown.

Despite differences in the alignment, the phylogenetic trees based on the two core alignments produced identical topologies with similar Bayesian support values (Figure 6). The common tree united cv-lysozyme 1, cv-lysozyme 3, and a cDNA of unknown function from *C. gigas *(referred to here as cg-lysozyme 4) with a clade of lysozymes that includes the digestive cv-lysozyme 2 with strong support (BPP ≥ 0.99). The defensive cv-lysozyme 1 is positioned basally, with a well-supported cv-lysozyme 3/cg-lysozyme 4 clade coming off next as the sister to the clade including cv-lysozyme 2. The maximum likelihood tree (not shown) did not differ significantly from the Bayesian tree, with the only topological differences being the monophyly of *Calyptogena *and whether the *Tapes/Calyptogena *or *Cg-1/Ostrea *clade is sister to the crown group including cv-lysozyme 1, although support values for this tree are lower than for the Bayesian tree (Figure 6).

Although the function of i-type lysozymes from most other bivalves has not been demonstrated empirically, their isoelectric point and number of trypsin cut sites, mapped on the inferred phylogenetic tree, show a shift consistent with a transition from basal defensive function (with higher isoelectric points and more trypsin cleavage sites) to primarily digestive function (with lower pIs and fewer trypsin cleavage sites) in the clade including cv-lysozyme 2 (Figure 6). Cv-lysozyme 3 and its apparent ortholog cg-lysozyme 4 are unusual (compared to vertebrate lysozymes, e.g. [10]) in possessing a low pI and relatively few trypsin cleavage sites, despite a defensive function. The tree topology is consistent with cv-lysozyme 3 and its *C. gigas *ortholog representing a preserved transitional form between the ancestral defensive function within *C. viriginica *and a derived digestive one.

**Figure 7:**
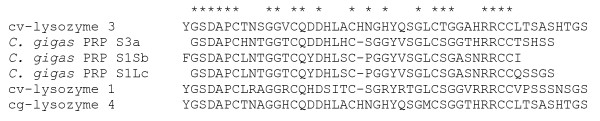
**Partial sequence alignment of cv-lysozyme 3 and 1 and the Pacific oyster peptidoglycan recognition proteins**. The unique stretch of amino acid residues from the N-termini of cv-lysozyme 3 and 1(see Figure 5) was aligned with a repeat motif from peptidoglycan recognition proteins (PGRPs) of the Pacific oyster *C. gigas*. ^a^BAG31899, ^b^BAG31896, ^c^BAG31897.

The full BAli-Phy alignment revealed a stretch of 48-49 amino acid residues with strong similarity between cv-lysozyme 1, cv-lysozyme 3, and cg-lysozyme 4 that was not seen in any other bivalve lysozyme. This unique amino acid sequence was compared to existing databases using BLASTp to ascertain its possible relation to other known domains. The search obtained three significant hits (Figure 7), all to repeat motifs from peptidoglycan recognition proteins (PGRPs) of *C. gigas*. Cv-lysozyme 3 aligned with residues from a similar position near the N-termini of these three PGRPs (Figure 7) and also to two additional repeats that followed in each soon after in each PGRP. Maximum similarity between cv-lysozyme 3 and one of the PGRP repeats was nearly 70% (30/43 residues in common, or 69.7%).

### Positive election on i-type lysozymes in bivalve molluscs

Values for ω (the ratio of dN, the number of nonsynonymous substitutions per nonsynonymous site, to dS, the number of synonymous substitutions per synonymous site) were generally low (around 0.11, see Table 3), suggesting lysozymes have generally been subject to purifying selection. Two tests (H2 and H5), however, did result in ω > 1. Both of these allowed the branch (b in Figure 6) leading to the clade with the digestive cv-lysozyme 2 to have its own ω value. In both of these cases, ω was undefined due to a lack of synonymous substitutions. Inferred substitutions along branch b suggest a strong excess of amino acid changing substitutions over silent ones, around 14 to zero. These results are consistent with a burst of positively selected codon changes associated with the emergence of bivalve i-type lysozymes with a digestive function. These results, along with those for hypotheses H3 and H4, were significantly different from the null hypothesis of all branches having the same rate when tested with a χ^2 ^test with one degree of freedom.

**Table 3:** Positive selection tests.

Hypothesis	Change	Variation in ω	Background ω	Change ω
H0	None	none	0.111	n/a
H1	Episodic	branch a^† ^only	0.110	0.293
H2	Episodic	branch b^† ^only	0.110	undef (dN = 14.4, dS = 0)
H3*	shift	all in cv2C^‡^	0.090	0.154
H4*	shift	all in cv2C + branch b^†^	0.088	0.158
H5*	shift + episodic	all in cv2C, branch b^† ^different	0.087	undef (dN = 14.1, dS = 0)

* rate in test branch significantly different than background

† Branches a and b refer to Fig 6.

‡ cv2C = boxed cv 2 Clade in Fig 6.

Episodes or shifts in positive selection associated with the evolution of digestive function in bivalve i-lysozymes were tested.

## Discussion and Conclusions

The third lysozyme of *Crassostrea virginica*, cv-lysozyme 3, possesses a number of characteristics that mark it as an intermediary between two previously reported i-type lysozymes from this species. Cv-lysozyme 3 has a molecular mass and distinctive N-terminal amino acid sequence similar to cv-lysozyme 1, the lysozyme involved in the host defense [56,58]. Its chemical characteristics and major sites of gene expression, however, are more like those of cv-lysozyme 2, a lysozyme that functions in digestion [57]. In addition, the optimal muramidase activity of cv-lysozyme 3 was detected under pH and ionic strength conditions that favor the activity of neither cv-lysozyme 1 nor c-lysozyme 2. Consistent with this biochemical and expression characterization, our phylogenetic tree also placed the clade including cv-lysozyme 3 as the sister to a clade that includes cv-lysozyme 2. Moreover, an episode of positive selection was associated with the transition from defensive to digestive function.

Cv-lysozyme 3 shared properties with both cv-lysozyme 1 and cv-lysozyme 2 (Table 1). Specifically, cv-lysozyme 3 was composed of a similar number of amino acid residues and had a similar molecular mass as cv-lysozyme 1. It, along with its apparent ortholog cg-lysozyme 4, also shared a unique N-terminal domain with cv-lysozyme 1 that is absent in cv-lysozyme 2 and all other bivalve i-type lysozymes (Figure 5). On the other hand, the theoretical isoelectric point (pI), arginine residue number, and protease cutting sites in the amino acid sequence of cv-lysozyme 3 were closer to those of cv-lysozyme 2 [56-58]. Moreover, cv-lysozyme 3 and cv-lysozyme 2 are both expressed primarily in the digestive glands (Figure 4; [57]) whereas cv-lysozyme 3 minor expression sites overlapped with that of cv-lysozyme 1 (Table 1; [58]). These characteristics of cv-lysozyme 3 are consistent with the intermediate status of the new lysozyme between the previously reported cv-lysozyme 1 and cv-lysozyme 2.

The topology of our phylogenetic analysis is also consistent with the transitional status of cv-lysozyme 3 (Figure 6): cv-lysozyme 3 and its *C. gigas *ortholog branch off after the defensive cv-lysozyme 1 and as sister to a clade that includes the digestive cv-lysozyme 2. This suggests that the split between defensive lysozymes, like cv-lysozyme 1, and other lysozymes, including the digestive cv-lysozyme 2 and the potentially transitional cv-lysozyme 3, preceded the split of mytiloid and ostreid bivalves, a view consistent with taxonomic views that place these families (along with Chlamys of the Pectinidae) in the Pteriomorpha [63]. The sister group relationship of the digestive lysozymes (the clade containing cv-lysozyme 2 and the other digestive lysozymes, cv2C, of Figure 6) and the potentially transitional cv-lysozyme 3/cg-lysozyme 4 clade is consistent with an evolutionary history in which the latter are transitional forms that have been lost (or not yet found) in lineages outside of *Crassostrea*. An alternative in which cv-lysozyme 3 and cg-lysozyme 4 are not transitional forms but have instead arisen within the lineage leading to *Crassostrea *is not supported by the topology of phylogenetic tree in Figure 6, which places digestive lysozymes from *Mytilus *and *Chlamys *as closer to the digestive lysozymes from *Crassostrea *than the cv3/cg4 clade. However, the branch leading to the cv3/cg4 clade is not a long one, as would be expected for an early origin, so the possibility of a more recent genesis within *Crassostrea *cannot be entirely excluded. Finding a cv3 ortholog within mytiliods would resolve this issue. Expression levels of cv-lysozyme 3 appear to be low, suggesting that orthologs to this lysozyme may not yet have been found due to the rarity of their transcripts. We purified 18.6 mg of cv-lysozyme 1 from the first HIC peak in this research (not shown). Similarly, 2.2 mg of cv-lysozyme 2 can be purified from 5.5 g of crystalline style total proteins [57]. These qualitative data suggest that cv-lysozyme 3 expression level is far lower than the two other *C. virginica *lysozymes, such that the transcipts for this transitional form are rare. We predict that deeper sequencing of other bivalves with digestive lysozymes will reveal orthologs to cv-lysozyme 3. Alternatively, orthologs to cv-lysozyme 3 may have gone extinct in some bivalve lineages that once possessed them, including *Mytilus*.

Results from studies on c-type lysozymes in vertebrates and insects indicate that the shift from a defensive function to a digestive function is accompanied by changes from proteins that are basic (i.e., high pI) and sensitive to proteases (i.e., more protease cut sites in amino acid sequence) to acidic and more resistant to protease lysis [10,34-36,40]. This is also true of the two i-type oyster lysozymes, cv-lysozyme 1 and cv-lysozyme 2 [56-58]. Interestingly, the two non-oyster lysozymes (*T. japonica *in [51,64]; *Mytilus edulis *1 in [54]) biochemically determined to have high pI and numbers of trypsin cut sites (and thus inferred to have defensive function) occur basally in the phylogenetic tree, whereas the sole non-oyster lysozyme determined to be digestive based on low pI and few trypsin cut sites (*Chlamys islandica *in [52]) falls in the clade that includes the digestive cv-lysozyme 2. The single exceptional sequence within the digestive clade (with predicted high pI and many trypsin cut sites), cg-lysozyme 3, was generated from mantle tissue [65], and may represent an instance of reversal to the ancestral defensive function.

Despite their non-sister relationship, cv-lysozyme 1, cv-lysozyme 3, and cg-lysozyme 4 share a unique N-terminal domain. This suggests that this N-terminal domain may have been introduced on to the ancestral core of bivalve i-type lysozyme relatively recently. Itoh and Takahashi [66] identified a repeat domain that shows high sequence identity (> 50%) with the cv-lysozyme 1 N-terminal region at the N-terminus of some peptidoglycan recognition proteins (PGRPs) from the Pacific oyster, *C. gigas*. Our alignments (Figure 7) show an even higher level of sequence similarity between these PGRP repeats and the cv-lysozyme 3 and cg-lysozyme 4 N-terminal region. Because peptidoglycans constitute a major proportion of the bacterial cell wall, it may be reasonable to assume that this shared stretch of sequence is involved with the recognition and binding of bacteria in both PGRPs and lysozymes. A PGRP presumably involved in host defense exhibits the molecular signature of positive selection in ants [67]. Regardless of function, the unique N-terminal domain uniting cv-lysozyme 1, cv-lysozyme 3, and cg-lysozyme 4 apparently represents a modular sequence element that can be integrated into different proteins via domain shuffling [68]. Exon structure of bivalve lysozymes supports this possibility. An intron, which could facilitate domain shuffling, occurs just after the hard-to-align N-termini in the lysozymes of both *Mytilus edulis *1 and *Chlamys *[69,70].

Studies on c-type lysozymes of ruminant artiodactyls indicate that they include intermediate transitional forms between lysozymes that function in host defense and lysozymes that function in the stomach for digestion [71,72]. Ito et al [71], for example, reported that lysozymes purified from the kidney of cow and sheep have highest sequence identity with conventional defensive lysozymes, but show greater chemical similarity with stomach lysozymes (i.e., lowered pI and high enzymatic activity at low ionic strength). Some of these intermediates may have been retained because their chemical properties allow them to function under the pH and ionic strength conditions that are unfavorable for the other paralogs [71]. Interestingly, these intermediates of c-type lysozymes are also reported to have optimal pH and ionic strength conditions for lysozyme activity that differ from that of the lysozymes functioning in immunity [71]. Cv-lysozyme 3 showed a higher optimal pH for lysozyme activity compared to cv-lysozyme 1 and cv-lysozyme 2 (Figure 3; Table 1). Purified cv-lysozyme 3 also formed polymers, a phenomenon reported in different lysozymes [14,59-62], although cv-lysozyme 1 or cv-lysozyme 2 do not (not shown). Thus, cv-lysozyme 3 responds differently to pH and perhaps ionic strength than do other *C. virginica *lysozymes. The three *C. viriginica *lysozymes together would thus allow relatively high lysozyme activity to be maintained over a broad range of pH (5.5-9.0) and ionic strength (I = 0.005-0.26) (Figure 3B). The coexistence of three, or perhaps more, lysozymes with different chemical properties would thus help maintain a relatively high lysozyme activity level within tissues under the variable physiochemical conditions faced by oysters, which are osmoconformers and poikilotherms.

Cv-lysozyme 3 could potentially function both in immunity and digestion because it shares the properties of both lysozymes (i.e., cv-lysozyme 1 and cv-lysozyme 3). Previously, we purified just one lysozyme (i.e., cv-lysozyme 2) from the oyster crystalline style [57], indicating cv-lysozyme 3 is not secreted in quantity into the oyster digestive tubules, which will impede its function as a digestive enzyme. On the other hand, its amino acid sequence similarity with cv-lysozyme 1, including a shared unique amino-terminus (Figure 7), suggests a function in host defense, as the digestive gland can be an important portal of entry of pathogens.

The i-type lysozymes of bivalves share another similarity with c-type lysozymes in some vertebrate lineages: the evolution of digestive lysozymes from a defensive progenitor is accompanied by an episode of positive selection. Jollès et al [73] noted an elevated rate of nonsynonymous substitutions on the branch leading to a monophyletic clade of ruminant stomach lysozymes. Messier and Stewart [74] likewise found a burst of nonsynonymous substitutions on the branch leading to digestive lysozymes in Colobine monkeys. That burst involved an inferred 9 nonsynonymous changes, less than the estimated 14 for the branch leading to bivalve digestive lysozymes. Identifying which particular residues changed in bivalves, however, would be a far more speculative exercise than for Colobines, which diverged far more recently (15 My) than bivalve digestive lysozymes (which, given divergence times for the lineages leading to *Chlamys *and *Mytilus*, and *Crassostrea*, should have occurred in the Early Ordovician, about 480 My ago; [75]).

Considering together lysozymes that appear to be functional and phylogenetic intermediates in both ruminants and bivalves suggests an evolutionary pathway between ancestral defensive and derived digestive forms. The genes encoding lysozyme appear to have undergone multiple duplications within bivalves. This pattern is consist with that seen in the nematode genus *Caenorhabditis *[76], where different numbers of lysozyme paralogs among three congeners, along with varied phylogenetic relationships among them, suggest repeated cycles of gene duplication, divergence, and extinction. Duplication of an ancestral defensive paralog of lysozyme in bivalves would allow one copy to explore and adapt to physiological conditions from which the ancestral form was excluded. This specialization may be abetted by more tissue-specific expression of the duplicates [77]. Duplicates that were expressed in acidic environments, even while retaining their defensive function, would be fortuitously preadapted for digestive function, which would evolve following subsequent duplication of the intermediate form. This adaptive scenario makes specific predictions about the breadth of expression and physiological range of defensive, digestive, and intermediate forms (in those species that have the latter two). For example, physiological range should be broader and tissue-specificity should be lower in species having just a single (defensive) lysozyme. Lysozymes have already taught us much about adaptive protein evolution, and their further exploration should continue to inform how new protein functions evolve.

## Methods

### Cv-lysozyme 3 purification

Twenty liters of eastern oyster shell liquor were kindly provided by P&J Oyster Company in New Orleans, Louisiana, in March 2006. Shell liquor is a combination of shell cavity fluid and hemolymph released when oysters are shucked and has been found to contain lysozyme [78]. The shell liquor was freeze-dried and the powder resuspended in 1.2 L of deionized water by stirring overnight at 4°C. The suspension was then centrifuged at 3,200 g for 45 min at 4°C to remove the insoluble particles including hemocytes and tissue debris and the supernatant collected for lysozyme purification.

A combination of conventional ion exchange and size exclusion chromatographies, hydrophobic interaction chromatography (HIC), and reverse phase HPLC was used to purify cv-lysozyme 3. The shell liquor was first fractionated using the chromatographies with procedures modified from those used by Xue et al [56] for the purification of cv-lysozyme 1. The resulting fractions that contained lysozyme activity were further fractionated using hydrophobic interaction chromatography (HIC) with a HiPrep™ 16/10 phenyl FF (high sub) column (GE Healthcare Bio-Sciences Corp. Piscataway, NJ). Proteins loaded into the column were washed with a linear gradient from 1 M of ammonium sulfate water solution to deionized water at an elution rate of 5 mL/min for 40 min followed by an isocratic elution with deionized water for 10 min at the same elution rate. The elution was monitored for absorbance at 280 nm and tested for lysozyme activity.

We detected 2 absorbance peaks that contained fractions with high lysozyme activity. Because SDS-PAGE indicated that protein in the first peak was identical to the cv-lysozyme 1 purified previously [56], we took only the proteins collected in the second peak to HPLC purification. Reverse-phase HPLC purification was done using a Waters 600E system connected to a Waters Delta-Pak™ C_18 _column (15 μm particle size, 300Å pore size, 8 mm × 100 mm) (Waters Corporation, Milford, MA) with linear gradient elution. After loading of the protein sample, the column was eluted with a gradient from 20% acetonitrile containing 0.1% (v/v) TFA to 50% of acetonitrile containing 0.1% (v/v) TFA over 60 min at a flow rate of 1 mL/min. The elution was monitored for absorbance at 214 nm and each absorbance peak was collected as a single fraction. The fractions were freeze-dried to remove organic solvents and the proteins recovered in deionized water and stored at -20°C until use.

The purification was done at room temperature (20°C) and the purity of the resulting cv-lysozyme 3 was determined by SDS-PAGE using a 12% acrylamide/bis gel in Tris-HCl buffer, followed by staining of proteins in the gel with 1% (w/v) Coomassie blue R-250 in 10% (v/v) acetic acid-40% (v/v) methanol and destaining with 10% (v/v) acetic acid-40% (v/v) methanol solution. Protein concentration was measured by BCA using a Micro BCA Protein assay Reagent Kit (Pierce Biotechnology Inc, Rockford, IL) with bovine serum album as the standard.

### Lysozyme activity assay

Lysozyme activity was determined by monitoring the decrease in turbidity of a suspension of *Micrococcus lysodeikticus *(Sigma-Aldrich Corporation, St. Louis, MO) in an appropriate buffer solution as reported by Xue et al [56,57]. Buffer solutions were selected according to the requirements of the individual experiments (see below). The assay was done in a 96-well microplate where 20 μL of sample was mixed with 180 μL of a 0.8 mg/mL *M. lysodeikticus *suspension. The plate was measured at 25°C for absorbance at 450 nm for 5 min using a BenchMark Plus microplate reader (Bio-Rad Laboratories Inc, Hercules, CA) with a built-in kinetic mode. In this study, one unit of lysozyme was defined as the quantity that caused a decrease in absorbance of 0.001 unit/min using the *M. lysodeikticus *suspension prepared in 0.01 M MES-NaOH buffer, pH 6.0. All measurements of lysozyme activity were done in triplicate.

Optimal pH and ionic strength for lysozyme activity was determined using a set of 120 buffers covering pH range of pH 3.5-10.5 (3.5, 4.0, 4.5, 5.0, 5.5, 6.0, 6.5, 7.0, 7.5, 8.5, 9.5, and 10.5) and an ionic strength range of I = 0.005-2.60 (0.005, 0.010, 0.020, 0.040, 0.070, 0.100, 0.140, 0.180, 0.220, 0.260, and 0.280). The buffers were prepared with NaOH-acetic acid (pH3.5-5.5), Na_2_HPO_4_-NaH_2_PO_4 _(pH6.0-8.5), and boric acid-NaOH (pH9.5-10.5) as reported by Xue et al [56]. The results were expressed as percent activity with the highest activity detected was defined as 100%.

### Detection of antibacterial activities

To assess the antibacterial properties of the purified lysozyme, we measured the concentrations of cv-lysozyme 3 inhibiting the growth of *Escherichia coli *and *Vibrio vulnificus*. These bacteria were obtained from Dr. John Hawke or Dr. Marlene Janes at the Louisiana State University. Bacteria were grown in a nutrient broth containing 5 g beef extract, 2 g neopeptone, 0.1 g bactose dextrose, 1 g yeast extract and 10 g NaCl in 1 L of water and harvested in log phase. The bacteria were resuspended in phosphate buffered saline (PBS) at a density of about 10^6 ^bacteria per mL and 20 μL were added to 20 μL of twofold serially diluted lysozyme (200-6.25 μg/mL) in PBS or to 20 μL PBS alone (control) in 96-well plates. After 2 h incubation at room temperature, 160 μL of broth were added to each well and the plates were incubated at 30°C. The bacterium growth was measured at 570 nm after 12 h incubation with a BenchMark Plus microplate reader. Results were expressed as the minimum concentration (MIC) of lysozyme protein significantly inhibiting bacterial growth. All measurements were done in triplicate and the experiment was repeated twice. The data were analyzed by one factor ANOVA using the program SigmaStat (Systat Software Inc., Point Richmond, CA). A SNK's multiple comparison of means was performed when significant differences were found (P < 0.05).

### N-terminal sequencing

Purified cv-lysozyme 3 was separated by SDS-PAGE in a 12% acrylamide/bis gel under reduced conditions and transferred to a Sequi-blot™ PVDF membrane in 0.01 M CAPS-10% (v/v) methanol, pH 11, using a Mini Tran-Blot Electrophoretic Transfer Cell (Bio-Rad Laboratories Inc, Hercules, CA). The membrane was stained with 1% (w/v) Coomassie blue R-250 in 10% (v/v) acetic acid-40% (v/v) methanol and destained with 10% (v/v) acetic acid-40% (v/v) methanol solution. The PVDF membrane bearing the cv-lysozyme 3 was cut and washed 6 times in deionized water. N-terminal sequencing was done with automated Edman degradation using an Applied Biosystems Procise 494/HT protein sequencer (Applied Biosystems, Foster City, CA) at the Protein Chemistry Laboratory of the University of Texas Medical Branch in Galveston, Texas.

### Mass spectrometric determination of molecular mass and amino acid sequence

An aliquot (1 μL) of purified cv-lysozyme 3 in deionized water was mixed 1:3 (v/v) with 3,5-dimethoxy-4-hydroxycinnamic acid (sinapinic acid, SA) MALDI matrix and spotted on a sample plate for molecular weight determination. MALDI spectra were acquired on an Applied Biosystems Voyager STR instrument operated in linear mode using hen egg white lysozyme (MH+ 14306.6) as an external standard.

Purified cv-lysozyme 3 (70 μg) was reduced with 50 μL of 6.6 mg/mL dithiothreitol in 6 M guanidine HCl, 1.5 M Tris, pH 8.4 (buffer A) at 37°C for 45 min followed by alkylation with 50 μL of 15 mg per mL of iodoacetamide in buffer A at 37°C for 30 min. For intact protein analysis, excess reagents were removed by elution over C_18 _packed tip (Agilent Cleanup tip). Reduced and alkylated protein was mixed with matrix and analyzed as above.

For amino acid sequence analysis, cv-lysozyme 3 from the reduction/alkylation mixture was purified using a PepClean C-18 spin column (Pierce) following the manufacturer's protocol. Eluate in 70% acetonitrile (2 × 20 μL) was dried by speed vac. Dried cv-lysozyme 3 was solubilized in 50 mM ammonium bicarbonate buffer, pH 7.8 (50 μL) and trypsin (6 μg) was added for microwave digestion (CEM Corp., Matthews, NC) for 10 min at 60°C. Tryptic peptides were desalted using C_18 _ZipTips (Millipore) and eluted with α-cyano-4-hydroxycinnamic acid (CHCA) MALDI matrix (saturated in 70% acetonitrile, 0.1% TFA) for direct MALDI MS/MS analysis (Applied Biosystems 4800 instrument). MALDI MS/MS analysis was employed without peptide separation to directly obtain sequence information on peptides observed by MALDI mass spectrometry. Tandem mass spectra were manually interpreted and compared to the predicted cDNA sequence for assignment.

### Cv-lysozyme-3 cDNA cloning and sequencing

Cv-lysozyme-3 cDNA was identified by 3'- and 5'- rapid amplification cDNA ends (RACE) using cDNA synthesized from hemocyte total RNA as template. An initial search in the GenBank sequence databases using the N-terminal sequence determined by Edman degradation of purified protein as query identified an eastern oyster EST (Accession Number CV087997) that encompassed the N-terminal sequence of purified cv-lysozyme 3. We performed 3'-RACE using the BD SMART™ RACE cDNA Amplification Kit (BD Biosciences, Palo Alto, CA) and a primer (Lyso3-3' in Table 4) designed from this EST sequence. The 3'-RACE product was analyzed electrophoretically in 2.0% agarose gel stained by ethidium bromide and purified using an Ultra Clean Gel Spin DNA Purification Kit (Mo Bio Laboratories, Solana Beach, CA). The purified products were then cloned in a plasmid vector, pCR 2.1-TOPO, of TOPO TA Cloning Kit (Invitrogen, Carlsbad, CA) and sequenced with 3130 Genetic Analyzer using Big Dye Terminator v.3.1 sequencing reagent (Applied Biosystems, Foster City, CA). To ensure the sequence authenticity, three clones were randomly selected for sequence determination. The 5'-RACE reactions were performed using an intragenic primer (Lyso3-5' in Table 4) designed from the sequence of the 3'-RACE products and the products cloned and sequenced as described for the 3'-RACE.

**Table 4:** Primers used in cv-lysozyme 3 cDNA cloning, and semi-quantitative RT-PCR

Primer	Usage	Direction	Sequence
Lyso3-3'	3'-RACE	-	5'- CTT ACG GAA GCG ATG CAC CCT GCA CGA AC-3'
Lyso3-5'	5'-RACE	-	5'- CCG GTA GAA AAT GAG CCG GTG TGG GAG GC-3'
Lyso3-F	RT-PCR	Forward	5'- GAG TTT AAA CTG AAC TTA AG-3'
Lyso3-R	RT-PCR	Reverse	5'- CGT GAA CTT ATG TTT TAT TG-3'
28S-F	RT-PCR	Forward	5'- GTT GAC GCA ATG TGA TTT CTG C-3'
28S-R	RT-PCR	Reverse	5'-TAG ATG ACG AGG CAT TTG GCT A-3'

### Tissue specificity of cv-lysozyme 3 gene expression

Hemolymph was withdrawn separately from the adductor muscle sinuses of 5 eastern oysters, 9-13 cm in shell length and collected from Barataria Bay, Louisiana in July 2005, using a 1 mL syringe and a 25G needle and immediately centrifuged at 800 *g *for 2 min at 4°C. Supernatants were removed and the hemocyte pellets transferred into RNAlater (QIAGEN, Valencia, CA). The right valve of each oyster was then removed and 5 mm^3 ^fragments of the digestive gland, mantle, gills, style sac-midgut, labial palps, and gonad were excised and immediately immersed in RNAlater. The samples were stored at 4°C until use.

Total RNA was extracted from hemocytes and tissue fragments of organs sampled from the 5 individual oysters using an RNeasy mini kit (QIAGEN) and treated with DNase to prevent DNA contamination. cDNA was synthesized from 1 μg per sample of total RNA using an Omniscript Reverse Transcript Kit (QIAGEN) supplemented with oligodT primer and RNase inhibitor (Invitrogen). The synthesized cDNA was then measured for cv-lysozyme 3 cDNA using semi-quantitative PCR, with the oyster 28 S rRNA as a reference for sample normalization. Semi-quantitative RT-PCR was done using a Gene Cycler (Bio-Rad Laboratories Inc, Hercules, CA) in a total reaction volume of 20 μL containing 0.1 μL of Taq DNA polymerase, 2 μL of 10 × PCR buffer, 1.6 μL of dNTP (TaKaRa, Kyoto, Japan), 0.6 μL of each primer at 20 μM, and 1 μL of synthesized cDNA. Primers used for cv-lysozyme 3 cDNA detection (Lyso3-F and Lyso3-R) and the 28 S rRNA cDNA detection (28S-F and 28S-R) were shown in Table 4. The cv-lysozyme 3 primers were designed to amplify a PCR product of 631 bp that covered the entire cDNA excluding the polyA tail. PCR cycle numbers were optimized separately for cv-lysozyme 3 and 28 S rRNA to ensure the reactions were terminated within the linear non-saturated PCR phase. The final cycling conditions were as follows: an initial denaturation at 94.0°C for 10 min, followed by 35 cycles (for cv-lysozyme 3) or 25 cycles (for 28 S rRNA) of 94.0°C for 30 sec, annealing at 52.0°C for 30 sec, extension at 72.0°C for 30 sec, and a final extension at 72°C for 7 min. Ten microliters of the PCR products were separated in 2% agarose gel stained by ethidium bromide. The gels were then imaged using a Gel Doc™ XR system and analyzed using the Quantity One^® ^1-D analysis software (Bio-Rad Laboratories Inc.). The image analysis was performed by measuring the average optical intensity and area of each PCR product band followed by calculation of the band volume by multiplying the average optical intensity with the correspondent area. The cv-lysozyme 3 band volume was then divided by the 28 S rRNA band volume generated from a same sample and the ratio was defined as the cv-lysozyme 3 expression level of the correspondent tissue. The expression data were analyzed by one factor ANOVA using the SigmatStat software (Systat, Point Richmond, CA). A SNK's multiple comparison of means was performed when significant differences among organs were found (P < 0.05). The results were reported as mean ± standard deviation (SD) of 5 individual oysters.

### Computational sequence analysis

Amino acid sequence deduction of cv-lysozyme 3 cDNA sequence was performed using the software Genetyx Mac Ver. 10.1.6 (Software Development, Tokyo, Japan). Signal peptide was predicted by both neural networks and hidden Markov models on SignalP 3.0 server [79]. Sequence similarity was determined using BLAST program on the server of National Center of Biotechnology Information (NCBI) [80,81]. Molecular weight, isoelectric point (pI), and protease cleavage sites were predicted using ProtParam and PeptideCutter on the ExPASY Server [82].

### Alignment and phylogenetic analysis of sequences

If cv-lysozyme 3 represents a preserved transitional form between the ancestral defense function of lysozyme and its presumably derived digestive function, then cv-lysozyme 3 and its orthologs should fall phylogenetically as the sister group to the digestive lysozymes. To test this hypothesis, we preformed a phylogenetic analysis of known bivalve i-type lysozymes (Table 5), using sequences from two annelids (*Eisenia andrei *and *Hirudo medicinalis*) as outgroups.

**Table 5:** I-type lysozymes identified from bivalve mollusc species used for phylogenetic and positive selection analyses.

Species	GenBank Accession	Reference
*Bathymodiolus azoricus*	AF334663	[70]
*B. thermophilus*	AF334664	[70]
*Crassostrea virginica 1*	AB206328	[58]
*Crassostrea virginica 2*	AB252064	[57]
*Crassostrea virginica 3*	AB427186	This paper
*Calyptogena sp. SB2001_1*	AF334666	[70]
*Calyptogena sp. SB2001_2*	AF334667	[70]
*Crassostrea gigas 1*	AB179775	[96]
*Crassostrea gigas 2*	AB288344	[97]
*Crassostrea gigas 3*	AB307634	[65]
*Crassostrea gigas 4*	CU994883	Favrel, direct submission
*Chlamys islandica*	AJ250028	[52]
*Eisenia andrei*	DQ339138	Joskova et al. direct submission
*Hirudo medicinalis*	U24122	[98]
*Mytilus edulis 1*	AF334662	[70]
*Mytilus edulis 2*	DQ268868	Caponera and Rawson direct submission
*Mytilus galloprovincialis 1*	AF334665	[70]
*Mytilus galloprovincialis 2*	AB298451	Itoh et al. direct submission
*Ostrea edulis*	AB179776	[96]
*Tapes japonica*	AB091383	[64]

Sequences from two annelids (*Eisenia andrei *and *Hirudo medicinalis*) were used as outgroups

The inferred amino acid sequences for bivalve lysozymes varied greatly in length, ranging from 116 (*Calyptogena *sp. 1) to 228 (*M. gallo*. 2) residues. Such length variation necessarily results in gaps in the alignments used for phylogenetic analysis. To minimize the impact of these gaps on the outcome of our analyses, we used two alignment algorithms (ClustalW and Bali-Phy) and excluded regions where only a small proportion (less than one-third) of sequences shared insertions. ClustalW [83] combines an empirical model of residue replacement with gap penalties for adding new gaps and extending existing ones. The algorithm is progressive: similar sequences are aligned first, with increasing distant ones added to this foundation. However, any mistakes made early on, either due to a faulty initial guide tree or simply misaligned regions, can become locked and bias the final multiple sequence alignment, a problem exacerbated when attempting to align highly diverged sequences [84]. BAli-Phy [85] avoids this problem by not conditioning on a single guide tree but instead finding the multiple alignment with the highest posterior probability by estimating the alignment and tree topology simultaneously using a Markov Chain Monte Carlo (MCMC) sampler.

We used the default settings for ClustalW (ver. 2.012) alignment. For Bali-Phy (ver 2.02) analysis, we used the Empirical-WAG substitution model with four gamma rate classes and the default indel model. By default, the MCMC sampler in BAli-Phy collects information after each iteration and runs indefinitely until stopped by the user. We chose when to do this by first determining when convergence had occurred through visual inspection of output using Tracer v1.4 [86] and by periodically running the full analysis on the output, as recommended by the authors. After convergence, the Markov chain was then allowed to run until the effective sample size from the Markov chain was equal to or greater than 100. The final output was analyzed, with all the samples before convergence discarded as burnin.

The resulting full alignments contained long stretches of novel residues at both the amino- and carboxy-termini of several proteins. Such novel sequences commonly are introduced by the incorporation of noncoding DNA or via the fusion of parts from different genes [87], resulting in regions between orthologs that are not homologous and thus inappropriate for phylogenetic analysis. To minimize the impact of these gaps, we removed from each alignment all positions in which inferred insertions were shared by less than one-third of the sequences, including all positions in front of a conserved intron [70] that may facilitate domain shuffling at the amino-end of the protein (see Discussion). As part of the full output analysis, BAli-Phy produces alignment uncertainty (AU) plots that reflect the confidence of each position in the alignment by using Bayesian posterior probabilities (BPP). For the trimmed alignment, all columns and positions in the AU plot had BPP > 0.99, with the exception of five positions, two of which were > 0.5 and the remaining > 0.7.

The two trimmed core alignments (Figure 5) were used for further phylogenetic analyses. ProtTest v2.0 [88] was used to choose the appropriate model of amino acid evolution. The WAG + a gamma distribution was selected using the corrected AIC. This model was implemented in MrBayes v3.1.2 [89]. Two MCMC searches consisting of 4 heated chains were run in MrBayes for 1,000,000 generations, sampling every 100. After discarding the first 2500 samples, output files were checked with Tracer and PMSF values were calculated using the SUMP command to check for convergence. Convergence appeared to have occurred, so an all-compatible tree was calculated in MrBayes after discarding the first 2500 trees as burnin. A maximum likelihood tree for the ClustalW alignment was constructed using GARLI v0.96b8-r601 [90] with the WAG amino acid substitution model with a gamma distribution. Default settings were used for the GARLI analyses and 100 bootstrap replicates were performed. The resulting 100 trees were summarized into a consensus tree using Dendro-Py [91].

The novel amino terminus of cv-lysozyme 1 has high sequence similarity (> 50%) with a repeat sequence of unknown function from a family of peptidoglycan recognition proteins from the Pacific oyster, *C. gigas *[65]. Such high similarity could result from domain shuffling, which would violate a fundamental underlying assumption of phylogenetic sequence analysis: that all positions share a common history. We explored this possibility by performing a BLASTp search on the 49 residues near the N-terminus of cv-lysozyme 3 that the BALi-Phy analysis could align only to a similar region in cv-lysozme 1. Only sequence hits with Z-scores ≥ 5 were considered.

### Testing for positive selection

Adaptive protein evolution leaves a signature of selection in the form of a high nonsynonymous (amino acid altering) substitution rate relative to the synonymous (silent) rate [92]. Values of ω less than one indicate purifying selection, while ω greater than one indicates positive selection. Modifications of this general approach [93] can be used to identify branches on a phylogenetic tree where selection shifts or where episodic change is concentrated. More specifically, these tests employ a maximum likelihood approach to estimate values of ω for specified partitions of branches and whether these values differ.

We used PAML v4.2a [94] to test for evidence of lineage-specific changes in selection pressure by differences in selection. Because comparisons of deeply divergent sequences can cloud the signal of selection [95], we removed six sequences that were distant from the clade containing the digestive lysozymes prior to the selection analysis (see Fig 6 for large box indicating the species and topology used for the analysis).

To test whether the shift from a defensive function in cv-lysozyme 1 to a digestive function in cv-lysozyme 2 was associated with either an episodic burst of adaptive sequence change or a shift in ω values, we tested six hypotheses (Table 5). First (H0), we assumed that ω was equal among all lineages of the lysozyme tree. Next, we tested for episodic burst of change, where ω increased either on the branch leading to cv-lysozyme 3 and the clade (cv2C) including the digestive lysozyme cv-lysozyme 2 (H1) or at the branch leading to the clade including digestive lysozyme cv-lysozyme 2 (H2). We tested for shifts in ω either for all of the digestive cv-lysozyme 2 clade (H3) or for the digestive cv-lysozyme 2 clade plus branch b leading to them (H4). Finally, we evaluated a combination model with an episodic burst at branch b and a different ω in the digestive cv-lysozyme 2 clade thereafter. The significance of any difference was tested using a χ^2 ^test with one degree of freedom.

## Authors' contributions

QGX and MEH equally contributed to this work; QGX designed and carried out the purification and characterization of the new eastern oyster lysozyme, analyzed amino acid sequence of the bivalve lysozymes, and, together with MEH, prepared the manuscript; MEH performed the phylogenetic and positive selection analyses, in addition to jointly preparing the manuscript; KLS analyzed the purified lysozyme protein using mass spectrometry; NI cloned and sequenced the cDNA of the new lysozyme; RIE did sequence alignments, phylogenetic tree inference, and positive selection test by using related computer programs; RKC was involved in the determination of cDNA sequence and the gene expression; JFL was Co-PI with QGX for the LSG project on the new lysozyme purification and characterization, and was involved in the characterization of the purified lysozyme. All authors read and approved the final manuscript.
